# Supplementary material for: Fault valving and pore pressure evolution in simulations of earthquake sequences and aseismic slip
Source: Nat Commun. 2020 Sep 24;11:4833. doi: 10.1038/s41467-020-18598-z (PMC7515873; doi:10.1038/s41467-020-18598-z)
Supplement: Supplementary file 1 — Supplementary Information [file 41467_2020_18598_MOESM1_ESM.pdf]

Supplementary Information for **Fault Valving and Pore Pressure Evolution in Simulations of Earthquake Sequences and Aseismic Slip** by Zhu et al.

Supplementary Figures

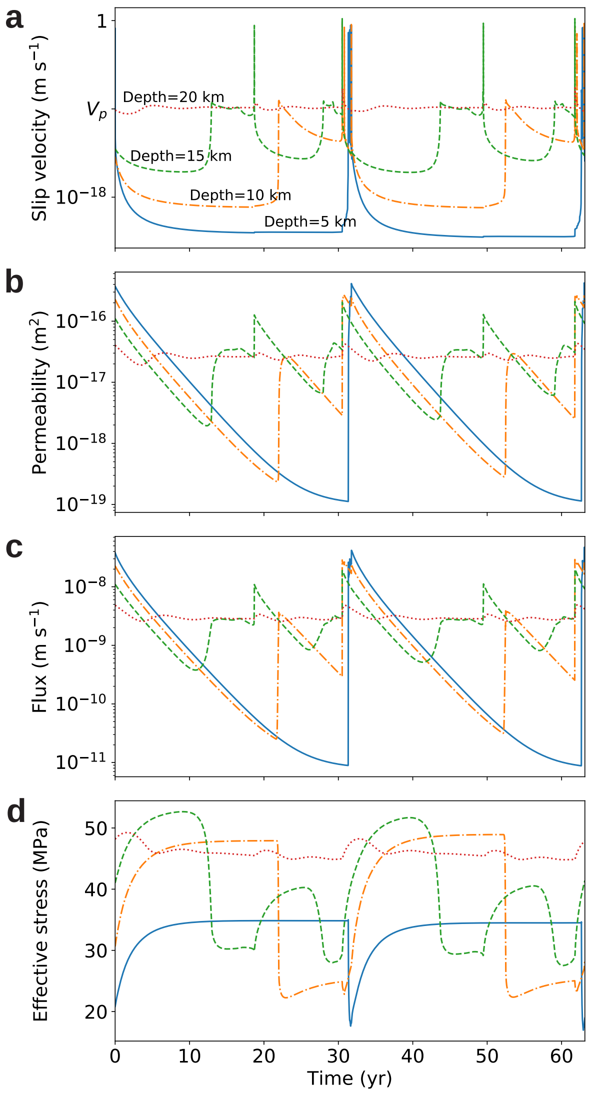

Supplementary Figure 1: History of fields at depths of 5, 10, 15, and 20 km, summarizing cyclic build-up and release of overpressure through upward fluid pulses in the high permeability state after earthquakes. Shown for $T = 3.17$ yr fault valving simulation.

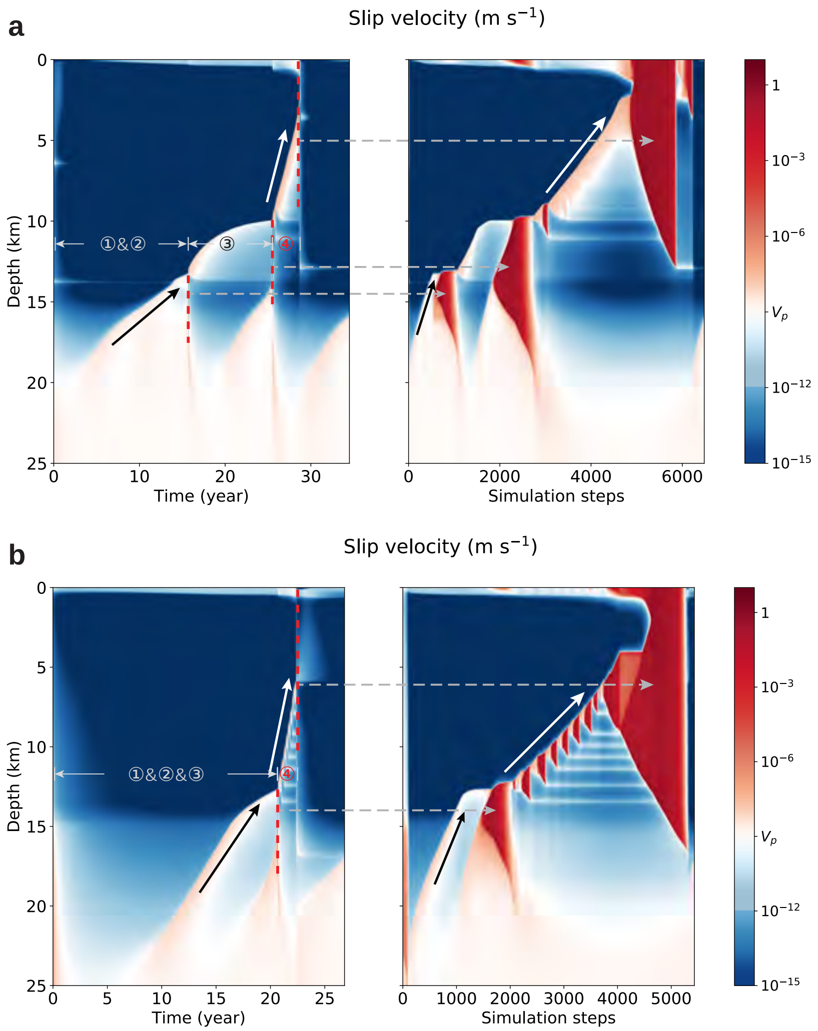

Supplementary Figure 2: Variations in the earthquake cycle for the $T = 3.17$ yr fault valving simulation featured in the main text, which has similar but not perfectly periodic behavior over each cycle. Time $t = 0$ in the plots always follows a large earthquake that ruptures most or all of the seismogenic zone, but is different for each cycle. **a**, **b** The ascent of the fluid overpressure pulse through the mid-seismogenic zone triggers slow slip rather than swarm-like seismic events. **c**, **d** The moderate-sized earthquake at the base of the seismogenic zone, marking the transition from phase 2 to 3, does not occur. Swarm-like seismic events occur in the mid-seismogenic zone.

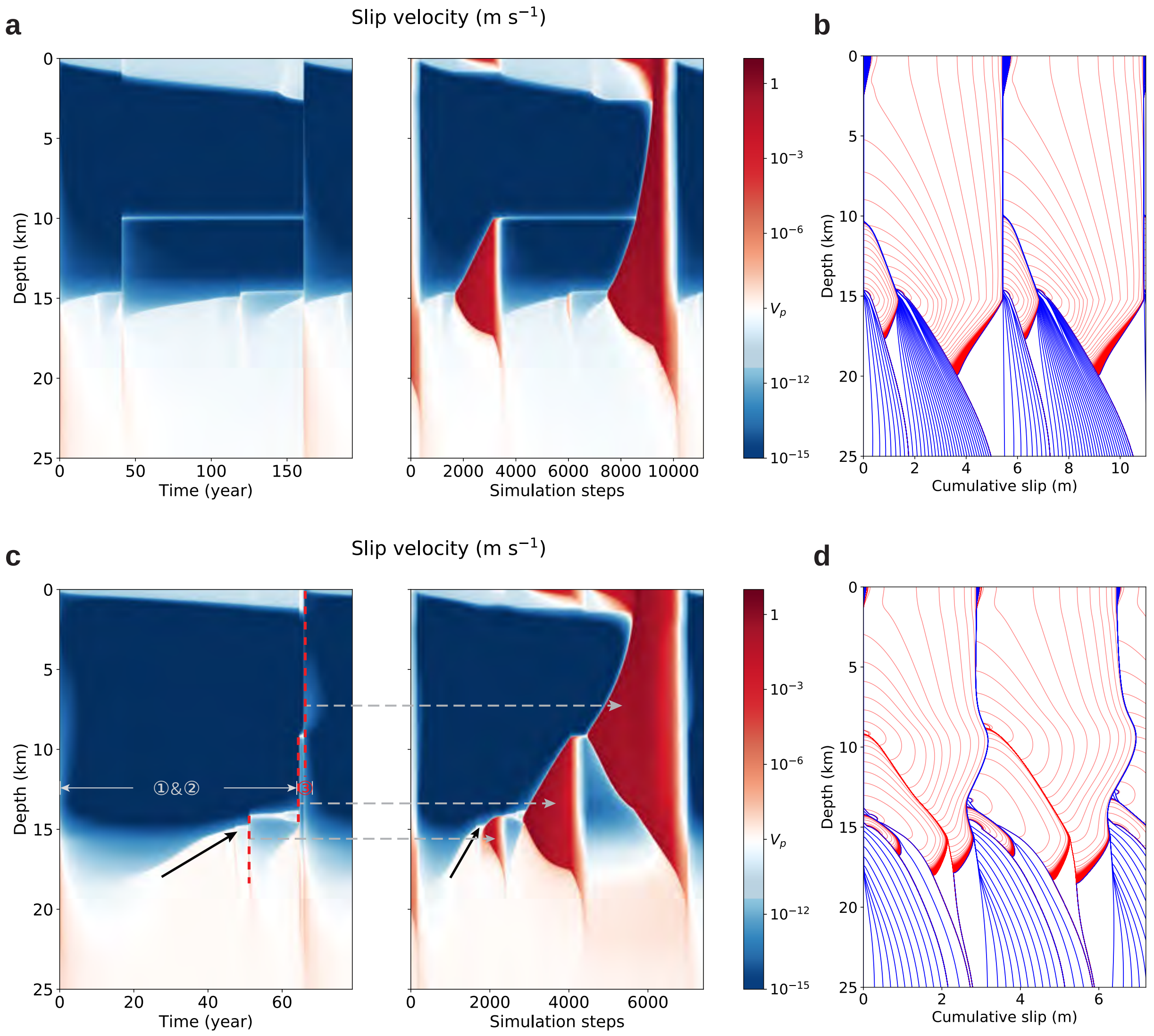

Supplementary Figure 3: Reference simulation with fixed p (top row, **a** and **b**) and fault valving simulation (bottom row, **c** and **d**) with $T = 31.7$ yr. Slip contours in **b** and **d** are plotted in blue every 4 yr for the interseismic period and in red every 1 s for the coseismic period.

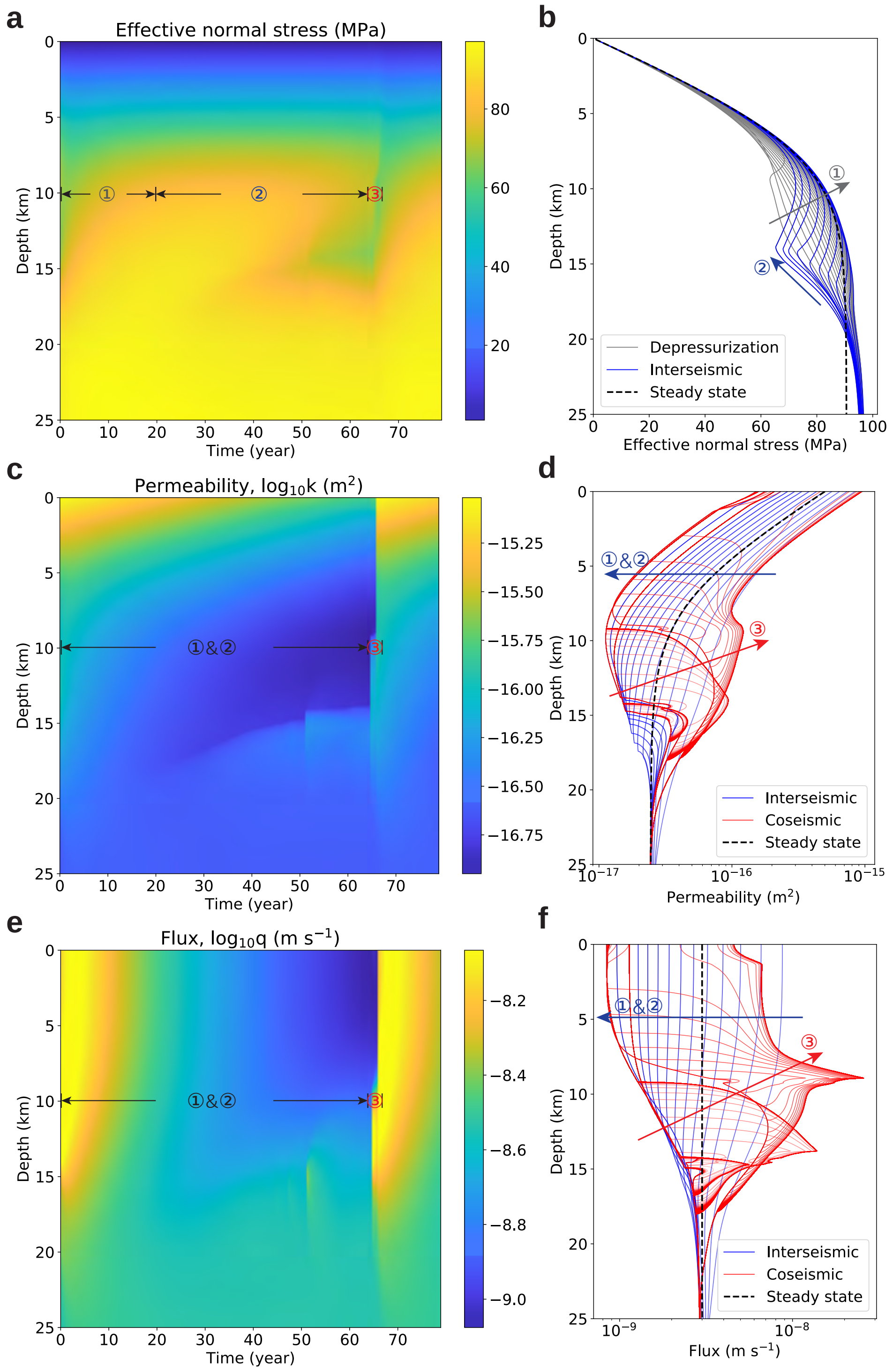

Supplementary Figure 4: Fault valving simulation with $T = 31.7$ yr. Contour intervals: blue, 4 yr; red, 1 s; gray in **b**, 1 yr. Steady state solution in dashed black lines.

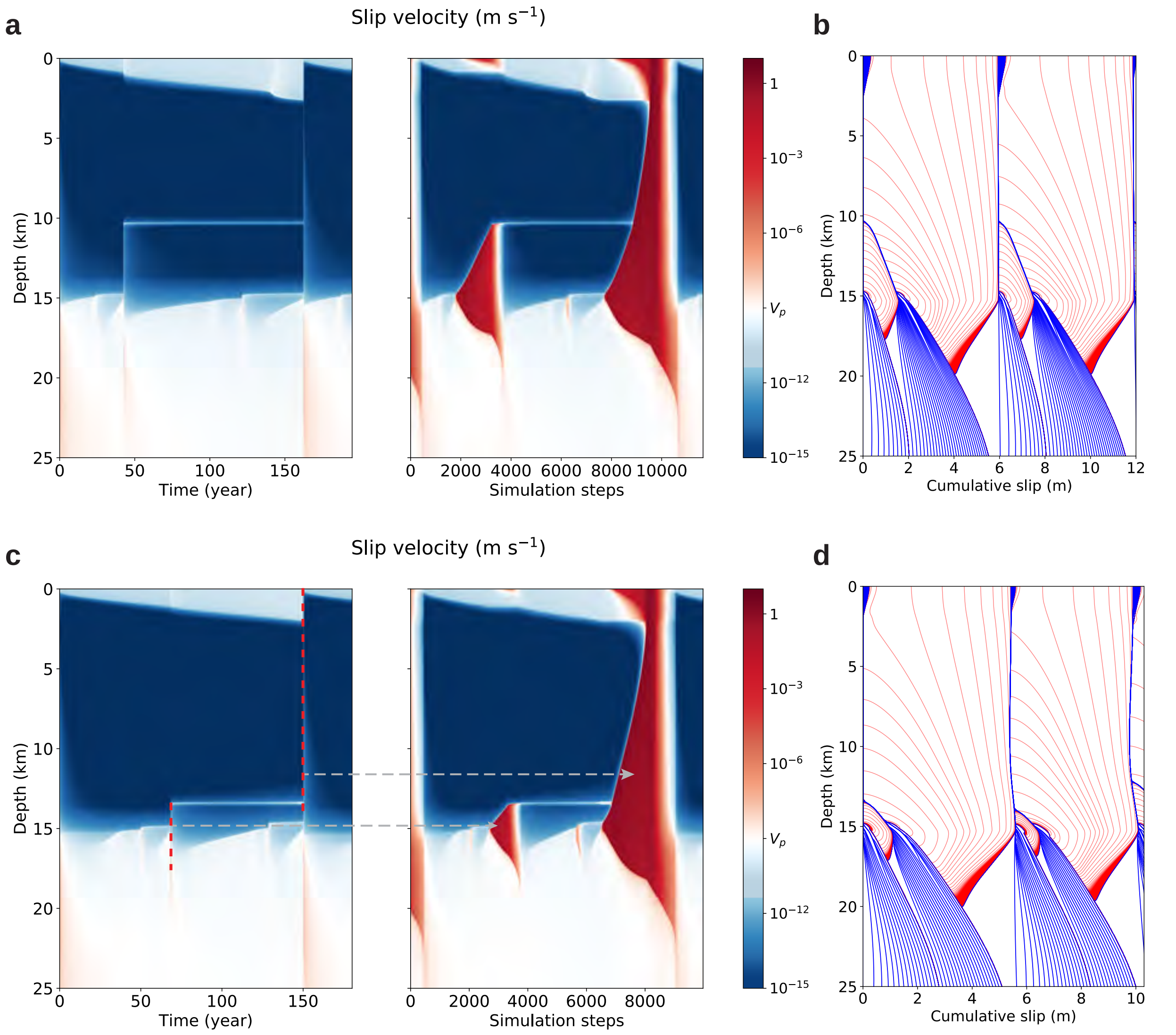

Supplementary Figure 5: Reference simulation with fixed p (top row, **a** and **b**) and fault valving simulation (bottom row, **c** and **d**) with $T = 317$ yr. Slip contours in **b** and **d** are plotted in blue every 4 yr for the interseismic period and in red every 1 s for the coseismic period.

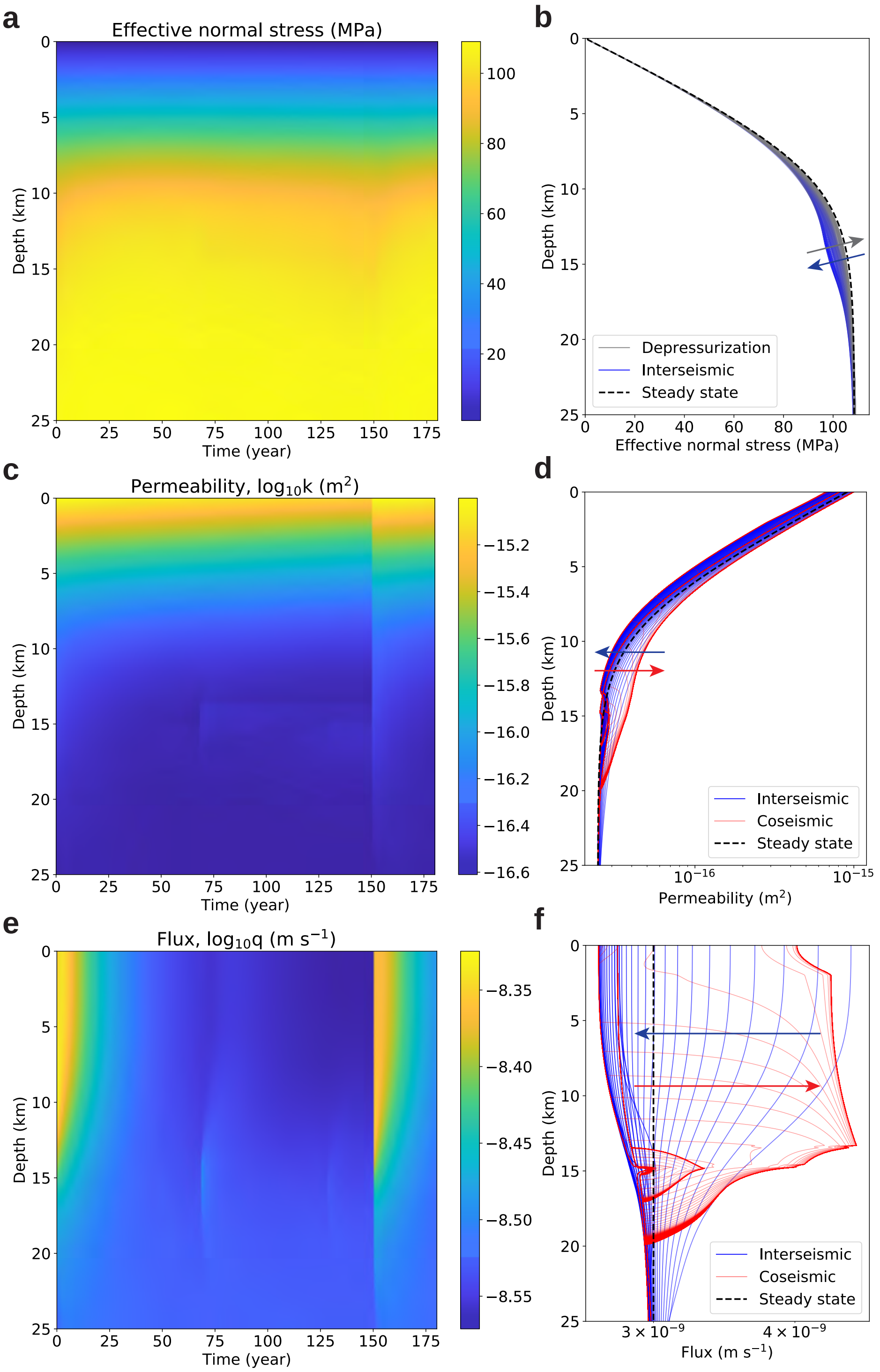

Supplementary Figure 6: Fault valving simulation with $T = 317$ yr. Contour intervals: blue, 4 yr; red, 1 s; gray in **b**, 1 yr. Steady state solution in dashed black lines.
