# Supplementary material for: Fault valving and pore pressure evolution in simulations of earthquake sequences and aseismic slip
Source: Nat Commun. 2020 Sep 24;11:4833. doi: 10.1038/s41467-020-18598-z (PMC7515873; doi:10.1038/s41467-020-18598-z)
Supplement: Supplementary file 2 — Description of Additional Supplementary Files [file 41467_2020_18598_MOESM2_ESM.pdf]

Description of Additional Supplementary Files

File name: Supplementary Movie 1
Description: Evolution of slip velocity and other fields for fault valving model with T = 3.17 yr

File name: Supplementary Movie 2
Description: Evolution of slip velocity and other fields for fault valving model with T = 0.317 yr
